# Integrated re-analysis of bulk and single-cell transcriptomic data reveals limited ferroptosis and neuroinflammation signatures after sevoflurane exposure in the aged rat hippocampus and human prefrontal cortex cells

**DOI:** 10.3389/fcell.2026.1938601

**Published:** 2026-09-29

**Authors:** Zhenrong Zhang, Chen Hu, Yan Liu, Qin Zhang, Jiafei Liu, Zhihua Sun

**Affiliations:** 1 Department of Anesthesiology, Ninghai First Hospital, Ningbo, Zhejiang, China; 2 Department of Anesthesiology, Huai’an Clinical Medical College of Jiangsu University (Huai’an Hospital of Huai’an City), Huai’an, Jiangsu, China; 3 Department of Anesthesiology, the 961st Hospital of the Joint Logistics Support Force of the Chinese People’s Liberation Army, Qiqihar, Heilongjiang, China; 4 Department of Anesthesiology, Xiangya Hospital, Central South University, Changsha, Hunan, China

**Keywords:** ferroptosis, hippocampus, neuroinflammation, oxidative stress, prefrontal cortex, sevoflurane, single-cell RNA sequencing, transcriptomics

## Abstract

**Background:**

Experimental studies have implicated ferroptosis in sevoflurane-associated neural injury, but the consistency of this signal across transcriptomic systems, species, and neural cell types remains uncertain. We therefore reassessed whether publicly available transcriptomic datasets support a coherent ferroptosis and neuroinflammation signature after sevoflurane exposure.

**Methods:**

We re-analysed two public transcriptomic datasets: GSE141242, comprising hippocampal tissue from aged rats exposed to 2.5% sevoflurane for 4 h (n = 3) or oxygen control (n = 3), and GSE196239, comprising human embryonic prefrontal cortex mixed-cell cultures exposed to sevoflurane, propofol, recovery conditions, or untreated control (one library per condition). After quality control, 67,272 single cells were retained. Bulk differential expression was assessed with an empirical-Bayes linear model, whereas the unreplicated single-cell conditions were summarized descriptively using key-gene expression, predefined gene-set scores, and observed cell-type composition. In addition, qRT-PCR was performed in C6 glioma cells and HA astrocytes assigned to Control, 3.3% sevoflurane for 6 h, H_2_O_2_ (200 μM, 24 h), or Sevo + H_2_O_2_ groups to compare ferroptosis-related gene regulation across the four conditions.

**Results:**

In the aged-rat hippocampus, no probe set reached FDR < 0.05 (minimum FDR = 0.071), and the two groups did not separate clearly in principal-component space. In the human prefrontal cortex cultures, selected ferroptosis-related genes showed only small descriptive differences between the single control and sevoflurane libraries, and predefined gene-set summaries were likewise similar. Observed cell-type proportions differed by less than two percentage points, and no broad cell type showed a coherent sevoflurane-associated ferroptosis or inflammation pattern. The datasets were compared qualitatively rather than by formal cross-species correlation because of their marked biological and replication differences. In the four-group qRT-PCR experiment, sevoflurane and H_2_O_2_ each reduced GPX4 and SLC7A11 and increased ACSL4 and PTGS2 in C6 and HA cells; the combined group showed the largest changes, and all treatment groups differed from Control (Dunnett-adjusted P < 0.05). Because the single-cell dataset contains only one library per condition, all between-condition single-cell comparisons are descriptive.

**Conclusion:**

The available public transcriptomic data do not provide robust evidence for a coordinated ferroptosis or neuroinflammation program attributable to sevoflurane across the examined systems. The four-group experiment showed that sevoflurane and H_2_O_2_ each regulated ferroptosis-related genes, with the largest changes in the combined group, providing complementary evidence of pathway perturbation under both exposures. Although the single-agent groups clarify the direction of each exposure-associated change, qRT-PCR alone cannot establish ferroptotic cell death or a pharmacologic interaction. Replicated studies with larger biological sample sizes and orthogonal ferroptosis assays are still required to establish whether ferroptosis is a reproducible component of sevoflurane-associated neurotoxicity.

## 1 Introduction

Ferroptosis is an iron-dependent form of regulated cell death characterized by phospholipid peroxidation and failure of cellular antioxidant defenses (Dixon et al., 2012; Stockwell et al., 2017; Jiang et al., 2021; Tang et al., 2019). The GPX4-glutathione axis is a central suppressive mechanism, whereas ACSL4-dependent incorporation of polyunsaturated fatty acids into membrane phospholipids can increase ferroptosis susceptibility (Yang et al., 2014; Doll et al., 2017). Ferroptotic cells may also release damage-associated molecular patterns, including HMGB1, thereby linking lipid peroxidation-driven cell injury with inflammatory signaling in some experimental contexts (Wen et al., 2019; Sun et al., 2020). These observations have motivated investigation of ferroptosis in neurological injury, but the presence and magnitude of ferroptosis remain highly dependent on cell type, model, and experimental conditions.

Sevoflurane is widely used for inhalational anesthesia, and experimental work has raised concern that prolonged or repeated exposure may alter neuronal and glial homeostasis, particularly in developing or aged nervous systems (Vutskits and Xie, 2016; Eckenhoff et al., 2020; Evered et al., 2018). More recent mechanistic studies have reported ferroptosis-related changes after sevoflurane exposure, including alterations in H2O2 accumulation, GPX4/SLC7A11 signaling, PRKCD-Hippo signaling, and PLIN4-associated pathways (Kang et al., 2024; Lv et al., 2024; Zeng et al., 2024). These studies support biological plausibility but use different species, developmental stages, exposure intensities, cell systems, and ferroptosis readouts. Accordingly, it remains uncertain whether a reproducible ferroptosis transcriptional signature is detectable across independent transcriptomic datasets.

Two public datasets provide complementary opportunities to examine this question. GSE141242 profiled hippocampal gene expression in aged rats after sevoflurane anesthesia and originally reported broad changes related to mitochondrial, metabolic, aging, and neurodegenerative processes (Wang et al., 2020). GSE196239 profiled human embryonic prefrontal cortex mixed-cell cultures by single-cell RNA sequencing after sevoflurane or propofol exposure and reported transient, cell-type-dependent transcriptomic responses (Chang et al., 2023). A recent secondary analysis of public bulk and single-cell datasets also proposed an SLC7A11-associated ferroptosis mechanism in sevoflurane neurotoxicity (Hu et al., 2024). These differing interpretations make transparent re-analysis, explicit control of expression scale, and careful distinction between biological replicates and individual cells particularly important.

Single-cell RNA sequencing can resolve cell-type-specific transcriptional states that are obscured in bulk tissue, but condition-level inference requires independent biological replication (Luecken and Theis, 2019; Squair et al., 2021; Crowell et al., 2020; Stuart et al., 2019). Treating individual cells as independent replicates can substantially inflate statistical significance when between-sample variability is not represented (Squair et al., 2021; Crowell et al., 2020). Cross-species interpretation also requires caution because homologous cortical cell types can retain broad cellular identities while differing considerably in gene expression, abundance, and molecular specialization between rodents and humans (Hodge et al., 2019). These issues are especially relevant here because the available datasets differ not only by species but also by brain region, developmental stage, culture context, exposure paradigm, and number of biological replicates.

The present study therefore focuses on a conservative question: whether these public datasets show a coherent and reproducible transcriptional pattern consistent with ferroptosis and neuroinflammation after sevoflurane exposure. Accordingly, we restrict single-cell comparisons to descriptive key-gene expression, predefined gene-set summaries, and observed cell-type composition; the two datasets are evaluated side by side rather than treated as formal cross-species validation. The cell experiment provides a complementary direct-exposure context in which C6 glioma cells and HA astrocytes were compared across Control, 3.3% sevoflurane for 6 h, H_2_O_2_ (200 μM, 24 h), and combined Sevo + H_2_O_2_ groups to assess the direction and magnitude of selected ferroptosis-related gene changes.

Specifically, we re-analysed the aged-rat hippocampal bulk dataset GSE141242 and the human prefrontal cortex single-cell dataset GSE196239, analysed the bulk data at the animal and probe-set levels, summarized the single-cell data descriptively by library and broad cell type, and examined selected ferroptosis-related transcripts across Control, sevoflurane-only, H_2_O_2_-only, and combined Sevo + H_2_O_2_ conditions in C6 glioma cells and HA astrocytes. Our objective was to determine the strength and limitations of the available evidence, rather than to infer a causal ferroptosis-neuroinflammation pathway or clinical biomarker model.

## 2 Methods

### 2.1 Data sources

Two public transcriptomic datasets were re-analysed. (1) GSE141242 (Affymetrix Rat Transcriptome Array 1.0, platform GPL22388): hippocampal tissue of 18-month-old male Sprague-Dawley rats exposed to 2.5% sevoflurane for 4 h (n = 3; GSM4199185-GSM4199187) or to 100% oxygen for 4 h (n = 3; GSM4199182-GSM4199184). The GEO Series Matrix reports RMA-normalized log2-scale expression, which was analysed without further transformation. (2) GSE196239 (10x Genomics Chromium v3, platform GPL24676): human embryonic prefrontal cortex primary mixed-cell cultures exposed to 3.3% sevoflurane (1.0 MAC) for 6 h, to propofol 20 µM for 6 h, or to the corresponding 2 h recovery conditions, together with an untreated control (one library per condition; GSM5866101-GSM5866105). Because each single-cell condition is represented by one library, all between-condition summaries are descriptive and individual cells were not treated as independent biological replicates.

### 2.2 Bulk microarray processing and differential expression

Probe-set-level RMA-normalized expression from GSE141242 was analysed on the reported log2 scale without additional transformation. The six animals were modelled with an empirical-Bayes linear model using limma with robust, intensity-trend variance moderation. The fixed contrast was Sevoflurane - Control, so positive log2 fold change denoted higher expression after sevoflurane. Benjamini–Hochberg correction was applied across 65,535 probe sets, and statistical significance was defined as FDR < 0.05.

### 2.3 Single-cell RNA-seq processing, clustering and annotation

Raw count matrices of GSE196239 were loaded per library (67,330 input cells). Cells were retained when they expressed 200–6,000 genes and had a mitochondrial fraction <20%; genes detected in fewer than 3 cells were excluded, leaving 67,272 cells and 23,597 genes. Data were normalized to 10,000 counts per cell and log1p-transformed; the top 2,000 highly variable genes were used for principal component analysis (PCA, 50 components), Harmony batch correction by library, a k-nearest-neighbour graph (30 PCs, 15 neighbours), UMAP embedding and Leiden clustering (resolution 0.8). Clusters were annotated by marker-based scoring against curated lineage sets; UMAP was used only for broad annotation and visualization, not to infer a treatment effect.

### 2.4 Descriptive single-cell summaries

Because each condition was represented by one library, no condition-level differential-expression P values were calculated and individual cells were not treated as biological replicates. Mean log1p expression was summarized for GPX4, SLC7A11, ACSL4, PTGS2, HMGB1, TFRC, and FTH1 by library and broad cell type. Predefined gene-set summaries were computed from mean log1p-normalized expression. The ferroptosis driver set comprised ACSL4, PTGS2, TFRC, ALOX5, LPCAT3, and HMOX1; the suppressor set comprised GPX4, SLC7A11, FTH1, FTL, SLC3A2, and GCLC. The inflammation set comprised HMGB1, S100A8, S100A9, IL1A, IL6, TNF, CXCL8, NLRP3, CASP1, C1QA, C1QB, TYROBP, AIF1, P2RY12. The ferroptosis contrast was driver minus suppressor. Cell-type composition was computed separately for each library; control-versus-sevoflurane comparisons were descriptive only.

### 2.5 Analysis scope across datasets

The bulk and single-cell datasets were evaluated side by side but were not subjected to formal cross-species correlation or joint significance testing. Because the datasets differ in species, brain region, developmental stage, tissue context, exposure conditions, and replication, interpretation focused on whether either dataset independently showed a coherent pattern in canonical ferroptosis-related genes and predefined gene-set summaries.

### 2.6 Quantitative RT-PCR validation

To examine regulation of key ferroptosis-related genes identified in the transcriptomic analyses, quantitative RT-PCR was performed in C6 rat glioma cells (ATCC CRL-2309) and HA human astrocytes (ATCC CRL-3050). Cells were cultured under standard conditions and assigned to Control, 3.3% sevoflurane exposure for 6 h, H_2_O_2_ treatment at 200 μM for 24 h, or combined Sevo + H_2_O_2_ groups. This balanced 2 × 2 design included both single-agent groups and the combined condition. Four ferroptosis-related genes were assessed: GPX4, SLC7A11, ACSL4, and PTGS2. GAPDH served as the internal reference. Relative expression was calculated using the 2^(-ΔΔCt)^ method. Three independent experiments were conducted. Effects were tested by two-way ANOVA (sevoflurane × H_2_O_2_) followed by Dunnett’s multiple-comparisons test against Control.

### 2.7 Statistical analysis and reporting units

Bulk analyses used individual animals (n = 3 per group), with FDR control across 65,535 probe sets. The single-cell cohort comprises five libraries (n = 1 per condition); all between-condition comparisons are descriptive. No cell-level inferential tests, cell-type-specific DEG counts, or enrichment analyses were used to support condition-level conclusions. Bulk modelling was performed in R (limma), and single-cell summaries in Python (scanpy, pandas, scipy); expression summaries used log1p-normalized rather than scaled values. For qRT-PCR, data are presented as mean ± SEM from three independent experiments; two-way ANOVA (sevoflurane × H_2_O_2_) was followed by Dunnett’s multiple-comparisons test against Control.

## 3 Results

### 3.1 Single-cell quality control

Across the five single-cell libraries, 67,330 input cells were reduced to 67,272 cells after quality control, with per-library retention of 11,616 to 11,603 cells (control), 13,910 to 13,894 (propofol 6 h), 13,297 to 13,286 (propofol recovery), 13,677 to 13,664 (sevoflurane 6 h) and 14,830 to 14,825 (sevoflurane recovery) (Figure 1A). Detected-gene counts, library sizes, and mitochondrial fractions were comparable across the observed libraries (Figures 1B–D). Detected-gene counts increased smoothly with library size (Figure 1E). QC retention was 99.9%–100.0% across all five libraries (Figure 1F).

**FIGURE 1:**
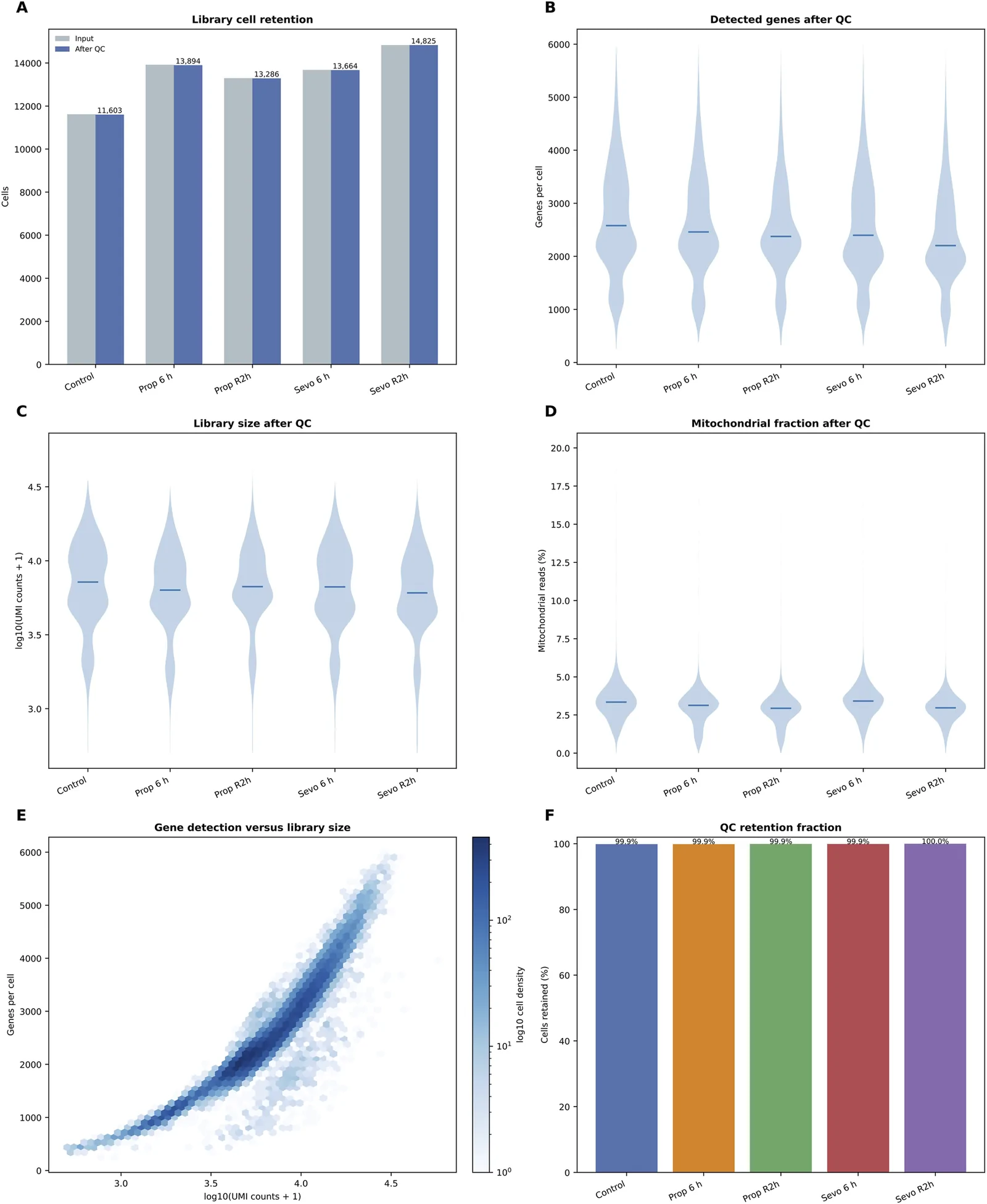
Single-cell quality control of GSE196239. **(A)** Input versus post-QC cell counts per library of GSE196239 (bars; values annotated). **(B)** Detected genes per retained cell by library (violin; median shown). **(C)** Library size after QC by library (log10 UMI counts + 1; violin; median shown). **(D)** Mitochondrial-read fraction per retained cell by library (violin; median shown). **(E)** Relationship between detected genes and library size (hexbin; colour bar, cell density). **(F)** QC retention fraction for each library (bars; values annotated).

### 3.2 Single-cell landscape and annotation of the prefrontal cortex cultures

Marker-guided clustering of 67,272 cells resolved nine broad states: Astrocytes (32,819), Oligodendrocytes (11,310), Interneurons (6,396), OPC (5,471), Proliferating cells (5,265), Immature neurons (3,252), Microglia (1,661), Stress-response glia (951), and Endothelial-like cells (147) (Figures 2A,F). The source libraries were intermingled in UMAP space; this view was used only to describe library distribution, not to infer a treatment effect (Figure 2B). Mean marker expression and marker detection frequency supported the nine broad labels (Figures 2C,D). Cluster-level marker-score separation provided an additional annotation audit (Figure 2E). Astrocytes were the largest observed population, followed by oligodendrocytes, while endothelial-like cells were rare (Figure 2F).

**FIGURE 2:**
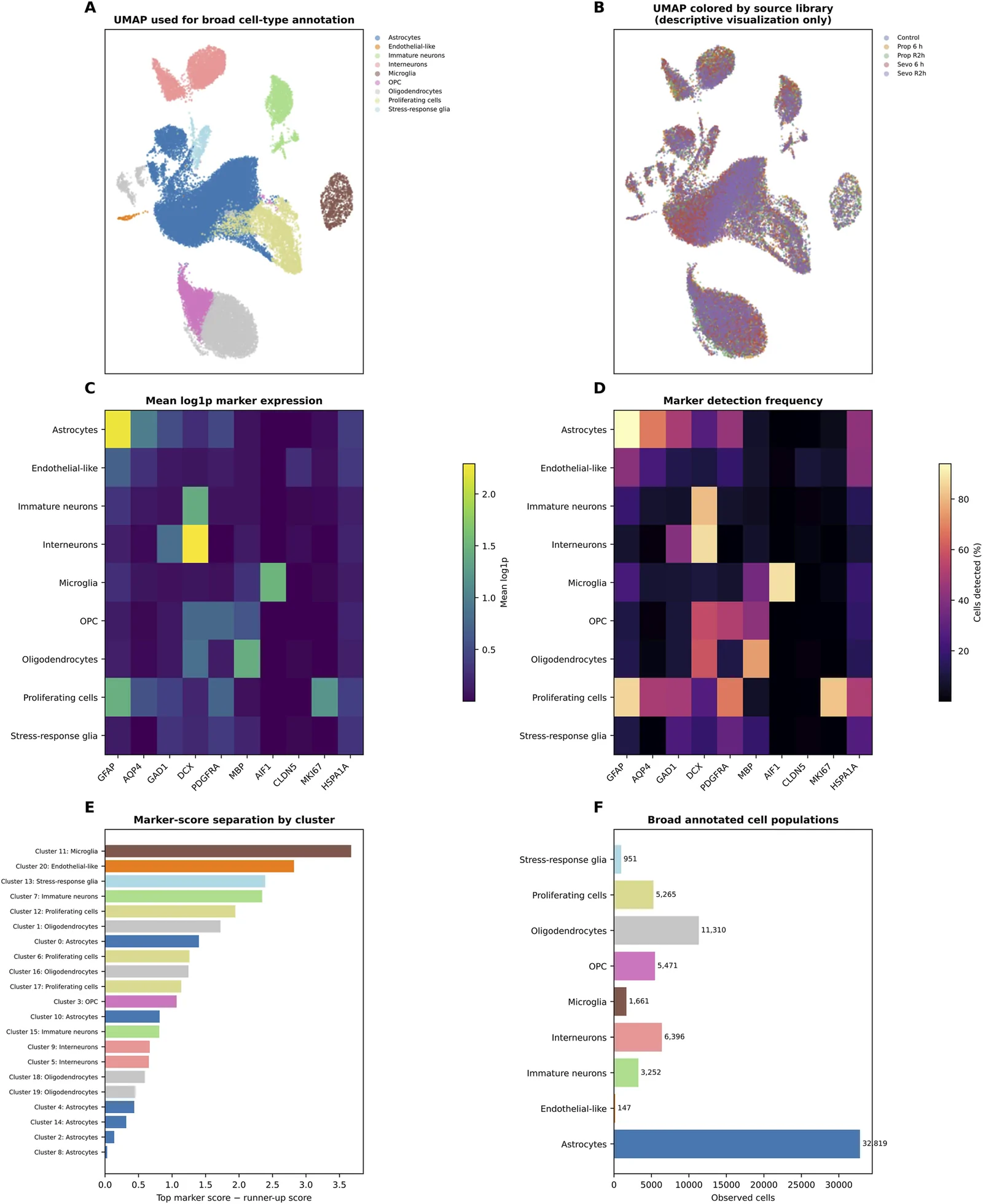
Broad cell-type annotation of GSE196239. **(A)** UMAP of 67,272 cells coloured by nine broad cell types. **(B)** UMAP coloured by source library; shown for descriptive visualization only. **(C)** Mean log1p expression of representative lineage markers by broad cell type. **(D)** Detection frequency of representative lineage markers by broad cell type. **(E)** Cluster-level separation between the top and runner-up marker scores. **(F)** Observed cell counts across the nine broad annotated populations.

### 3.3 Transcriptomic response to sevoflurane in the aged rat hippocampus

PCA of the six individual samples did not show clear treatment-driven separation (Figure 3A). Probe-set effect sizes were concentrated near zero (Figure 3B), with no intensity-dependent pattern in the MA plot (Figure 3C). Empirical-Bayes analysis identified no probe set at FDR < 0.05 among 65,535 tested probe sets; the minimum FDR was 0.071 (Figure 3D). The adjusted-P distribution remained above the significance threshold, and all top-ranked probe sets had FDR >= 0.071 (Figures 3E,F). Thus, the bulk dataset did not provide multiple-testing-adjusted evidence for a treatment-associated transcriptional program.

**FIGURE 3:**
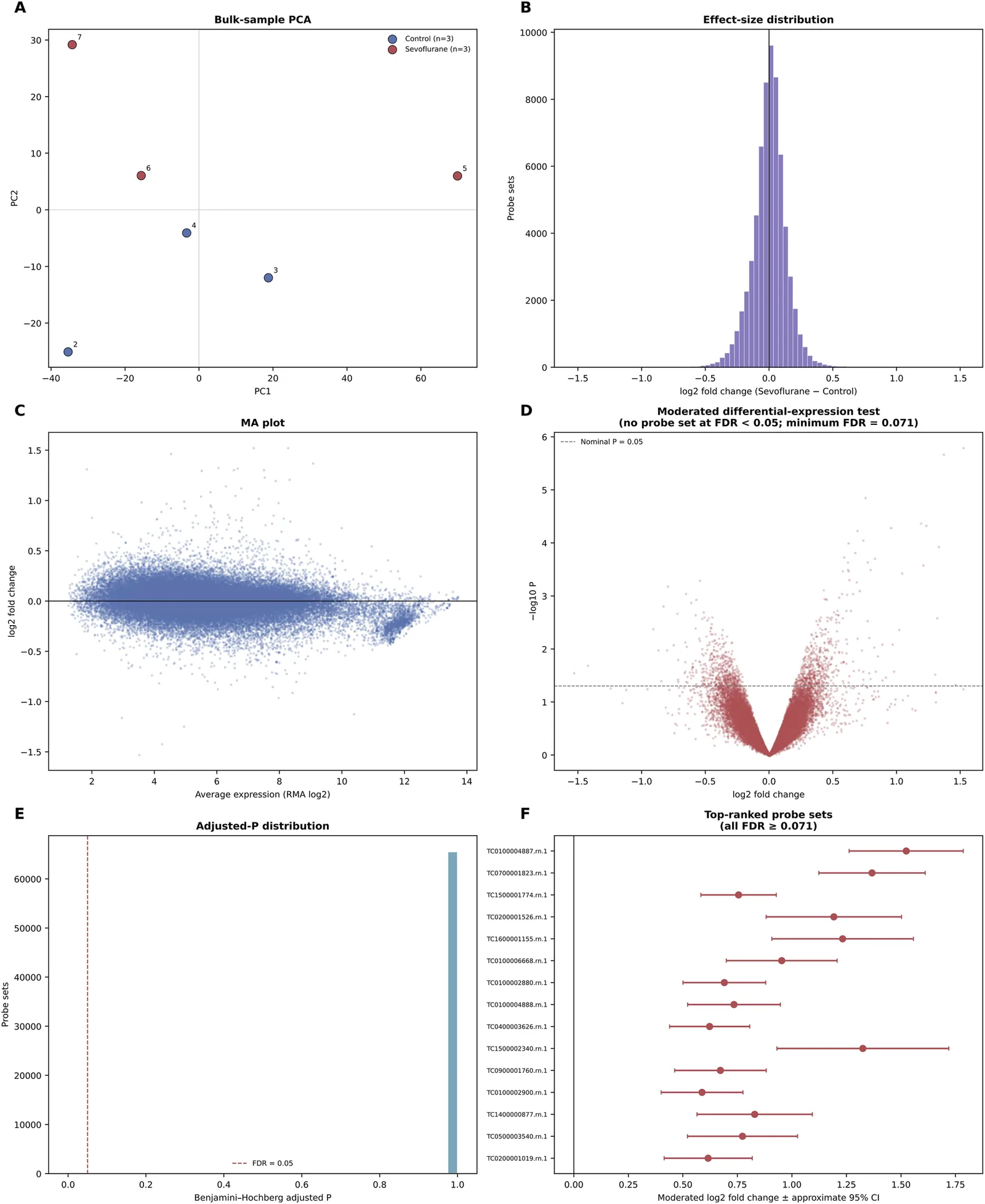
Bulk transcriptome of the aged rat hippocampus (GSE141242). **(A)** PCA of the six individual animals (n = 3 per group). **(B)** Distribution of probe-set log2 fold changes for Sevoflurane - Control. **(C)** MA plot of all 65,535 probe sets. **(D)** Volcano plot from the empirical-Bayes moderated analysis; no probe set reached FDR < 0.05 and the minimum FDR was 0.071. **(E)** Distribution of Benjamini–Hochberg adjusted P values; dashed line, FDR = 0.05. **(F)** Top-ranked probe-set effects with approximate 95% confidence intervals; all FDR ≥ 0.071.

### 3.4 Descriptive single-cell ferroptosis and inflammation summaries

In the single control and sevoflurane 6 h libraries, mean log1p expression differed only slightly for selected ferroptosis-related genes (Figure 4A). Mean log1p expression of selected inflammation-related genes was also similar between the two libraries (Figure 4B). For example, GPX4 was 2.034 versus 2.061, SLC7A11 0.273 versus 0.264, ACSL4 0.084 versus 0.083, PTGS2 < 0.001 in both, and HMGB1 2.001 versus 1.990. The ferroptosis gene-set contrast was similar across the five observed libraries (Figure 4C). The inflammation gene-set mean was likewise similar across libraries and showed no sevoflurane-specific increase (Figure 4D). UMAP mapping showed broad cell-identity-associated variation in the ferroptosis contrast (Figure 4E), while the inflammation gene-set mean was concentrated mainly in the microglial population (Figure 4F). Because each condition has one library, these comparisons are descriptive.

**FIGURE 4:**
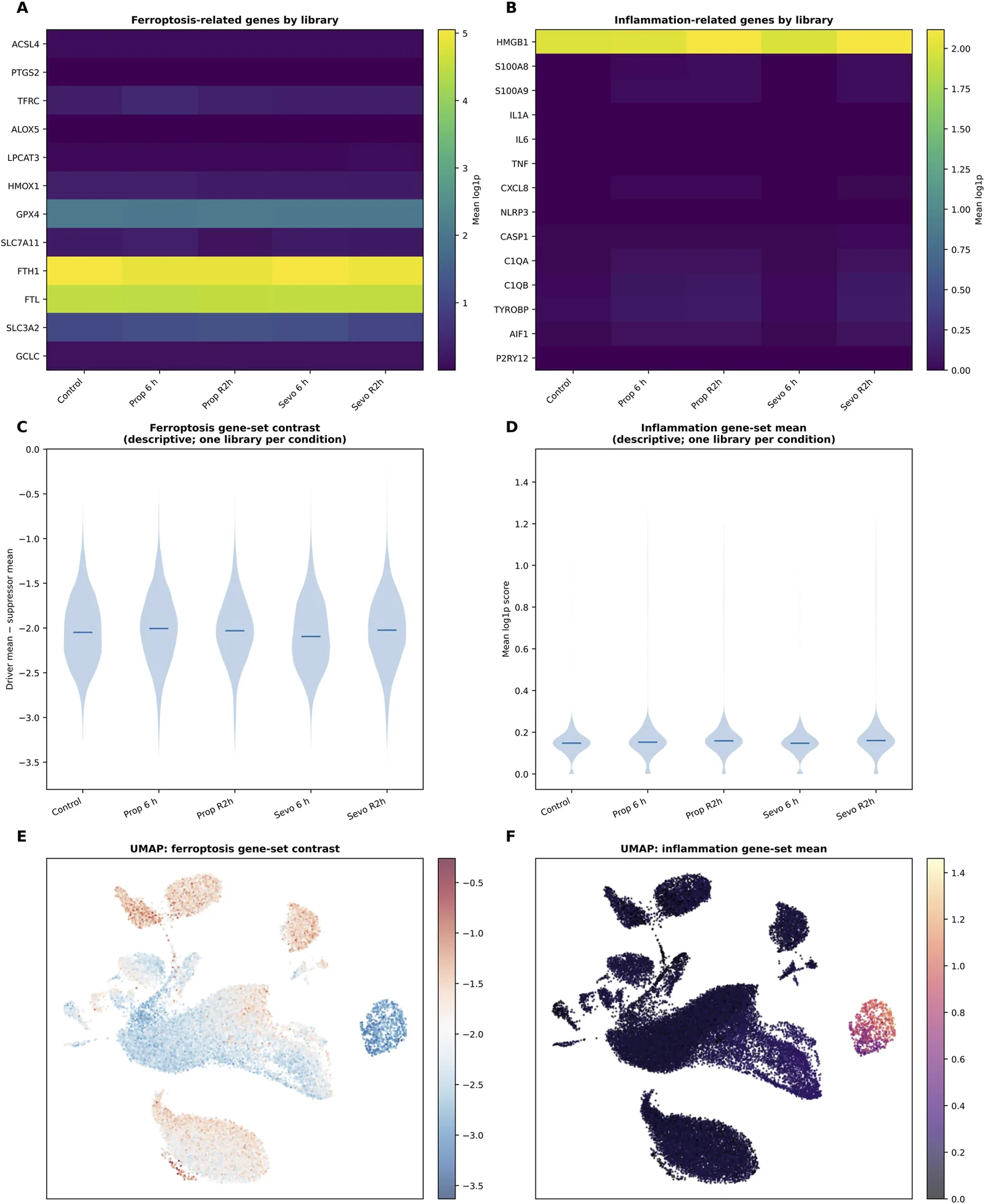
Descriptive library-level ferroptosis and inflammation summaries in GSE196239. **(A)** Mean log1p expression of ferroptosis-related genes across the five libraries. **(B)** Mean log1p expression of inflammation-related genes across the five libraries. **(C)** Ferroptosis gene-set contrast by library (driver mean minus suppressor mean; violin; median shown). **(D)** Inflammation gene-set mean by library (violin; median shown). **(E)** UMAP coloured by the ferroptosis gene-set contrast. **(F)** UMAP coloured by the inflammation gene-set mean. All between-library comparisons are descriptive because each condition contains one library.

### 3.5 Cell-type-specific descriptive ferroptosis summaries

Control and sevoflurane 6 h library means for ferroptosis-related genes were broadly similar within each broad cell type (Figures 5A,B). Cell-type-specific differences were small and mixed in direction (Figure 5C). Driver-gene means differed more across broad cell types than between the two observed libraries (Figure 5D), while suppressor-gene means showed the same general pattern (Figure 5E). The sevoflurane-minus-control difference in the ferroptosis contrast ranged from −0.089 to −0.001 across broad cell types (Figure 5F). These comparisons are descriptive because each condition contains one library; they do not identify a statistically responsive cell type.

**FIGURE 5:**
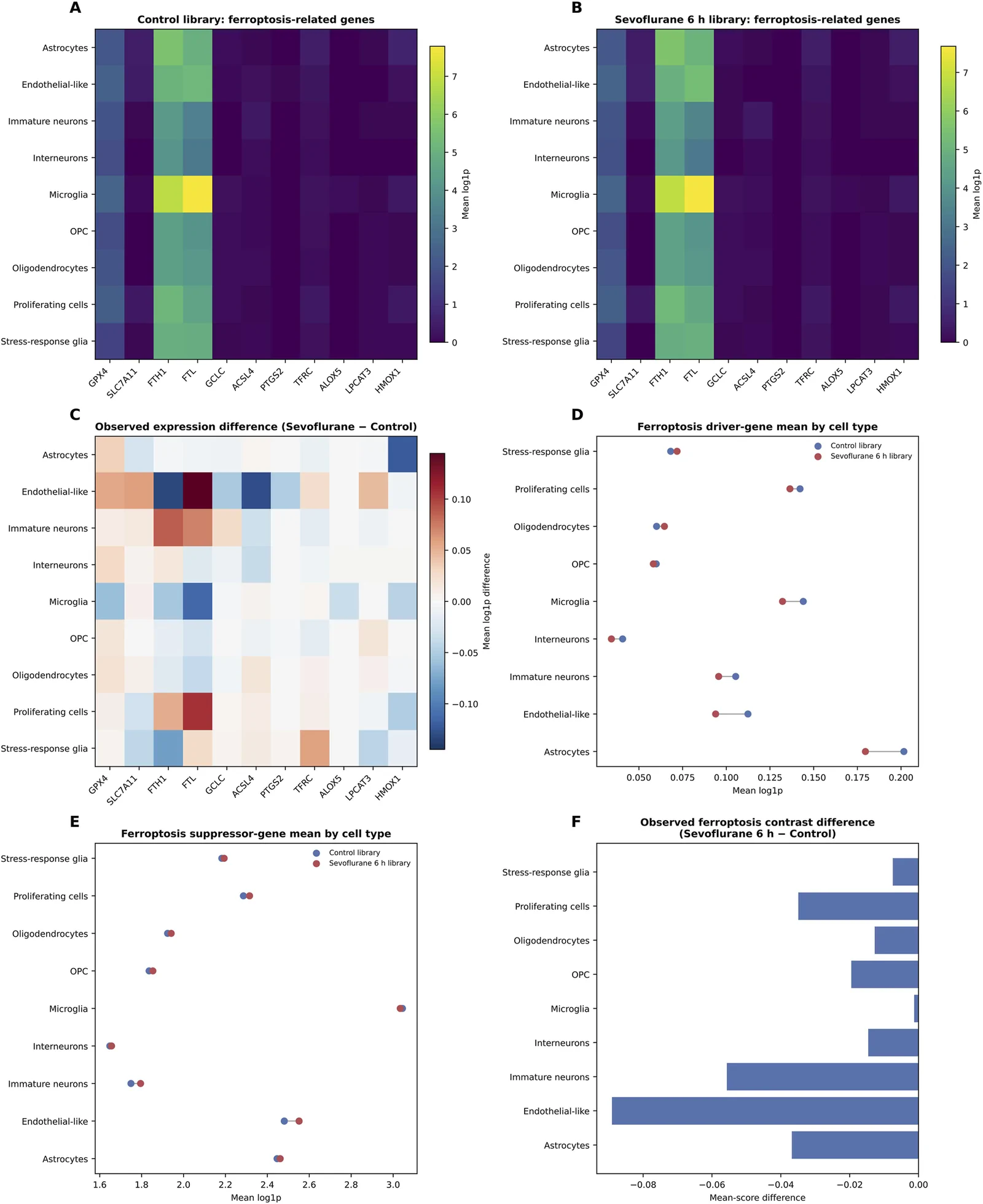
Cell-type-specific descriptive ferroptosis summaries for the control and sevoflurane 6 h libraries. **(A)** Mean log1p expression of ferroptosis-related genes by broad cell type in the control library. **(B)** Corresponding means in the sevoflurane 6 h library. **(C)** Observed mean-expression difference (Sevoflurane 6 h - Control). **(D)** Ferroptosis driver-gene mean by broad cell type in the two libraries. **(E)** Ferroptosis suppressor-gene mean by broad cell type in the two libraries. **(F)** Observed difference in the ferroptosis gene-set contrast. No panel represents a condition-level inferential test.

### 3.6 Cell-type-specific descriptive inflammation summaries

Control and sevoflurane 6 h library means for inflammation-related genes were broadly similar within each broad cell type (Figures 6A,B). Observed differences were small and mixed in direction (Figure 6C). Microglia showed the highest inflammation gene-set mean, but it did not increase in the sevoflurane 6 h library (Figure 6D). Across glial populations, inflammation-score trajectories did not show a sevoflurane-specific surge (Figure 6E). Detection-frequency differences for low-expression inflammatory genes were generally below one percentage point (Figure 6F). Together, these panels do not show a coherent inflammatory response attributable to sevoflurane.

**FIGURE 6:**
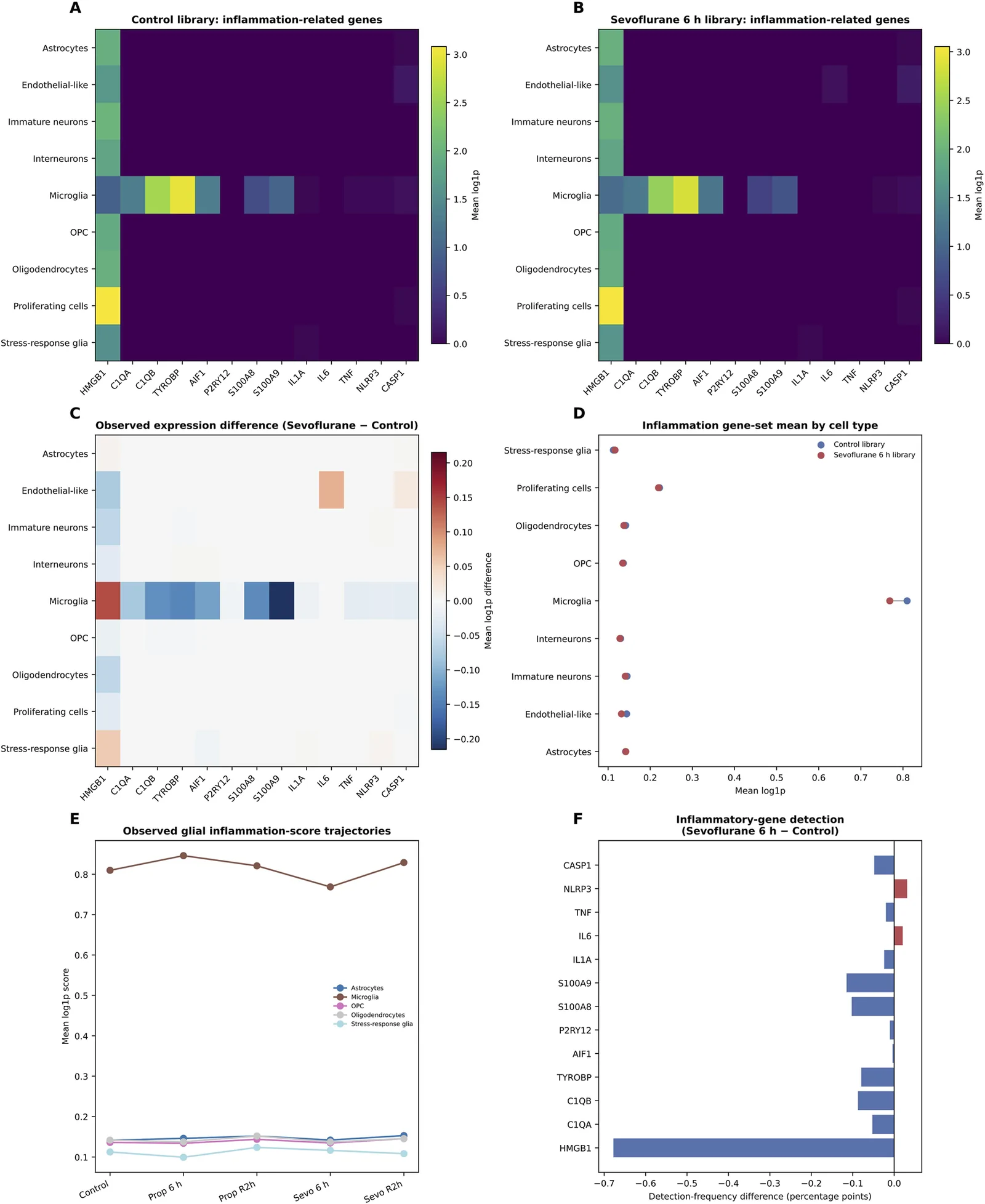
Cell-type-specific descriptive inflammation summaries for the control and sevoflurane 6 h libraries. **(A)** Mean log1p expression of inflammation-related genes by broad cell type in the control library. **(B)** Corresponding means in the sevoflurane 6 h library. **(C)** Observed mean-expression difference (Sevoflurane 6 h - Control). **(D)** Inflammation gene-set mean by broad cell type in the two libraries. **(E)** Observed inflammation-score trajectories in selected glial populations across all five libraries. **(F)** Detection-frequency difference for selected low-expression inflammatory genes (Sevoflurane 6 h - Control). All comparisons are descriptive.

### 3.7 Cell-type composition and expression-state dynamics

Observed cell-type composition varied modestly across the five libraries (Figure 7A), and the control versus sevoflurane 6 h comparison showed small lineage-specific differences (Figure 7B). Selected population trajectories differed modestly across libraries, with the largest sevoflurane 6 h versus control change remaining below two percentage points (Figures 7C,D). The underlying cell counts per library and broad type are shown in Figure 7E. Relative to control, percentage-point deviations across the other libraries remained modest and lacked a common lineage pattern (Figure 7F). Because each condition contributed one library, these shifts are descriptive.

**FIGURE 7:**
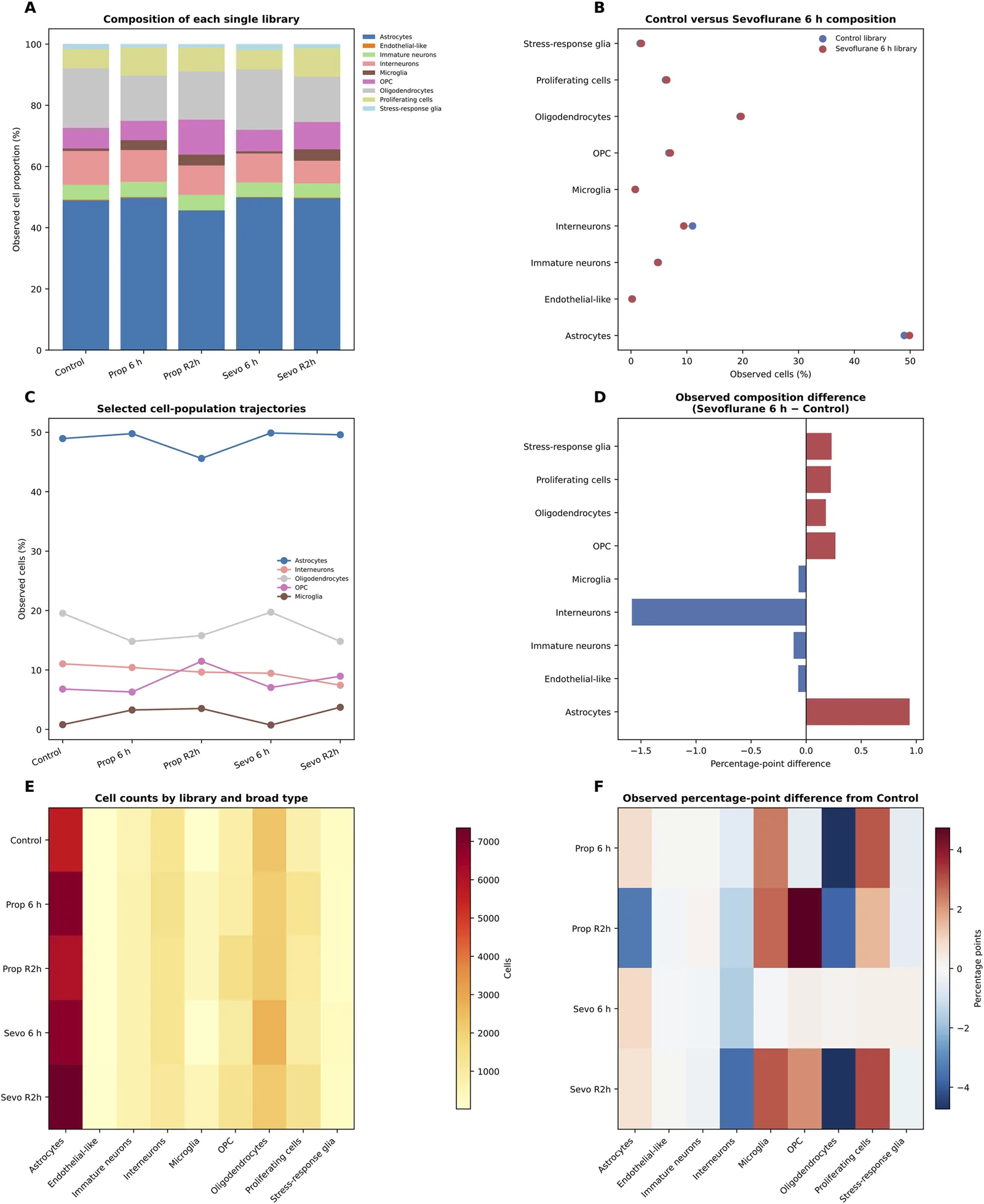
Observed broad cell-type composition across GSE196239 libraries. **(A)** Observed broad cell-type composition by library (stacked bars, %). **(B)** Control versus Sevoflurane 6 h composition dumbbell. **(C)** Observed trajectories of selected cell populations across the five libraries. **(D)** Composition difference in percentage points (Sevoflurane 6 h - Control). **(E)** Cell counts by library and broad cell type. **(F)** Percentage-point difference from Control for each of the other four libraries. All between-library comparisons are descriptive.

### 3.8 Integrated replication and selected-gene audit

Figure 8 summarizes the replication constraints: the bulk groups contain three animals each, whereas the control and sevoflurane single-cell conditions each contain one library (Figure 8A). Pearson correlations among the six bulk samples ranged from 0.981 to 0.995 without treatment-driven grouping (Figure 8B). Sample-level expression of GPX4, SLC7A11, ACSL4, PTGS2, HMGB1, TFRC, and FTH1 overlapped between the three control and three sevoflurane animals (Figure 8C). Moderated bulk effects for the selected genes had confidence intervals crossing zero, and none was significant after FDR correction (Figure 8D). Single-cell library means for the same selected genes were similar between control and sevoflurane 6 h (Figure 8E). Low-expression inflammatory genes were detected in fewer than 1% of cells in both libraries, limiting interpretation (Figure 8F). Collectively, neither dataset independently showed a coherent ferroptosis or neuroinflammation transcriptional program: the bulk analysis yielded no FDR-significant probe sets, while the unreplicated single-cell comparison showed only small descriptive differences in selected genes, gene-set summaries, and cell composition. Given these replication constraints, these observations are hypothesis-generating, and no causal claim about ferroptosis-driven neuroinflammation can be made from these data alone.

**FIGURE 8:**
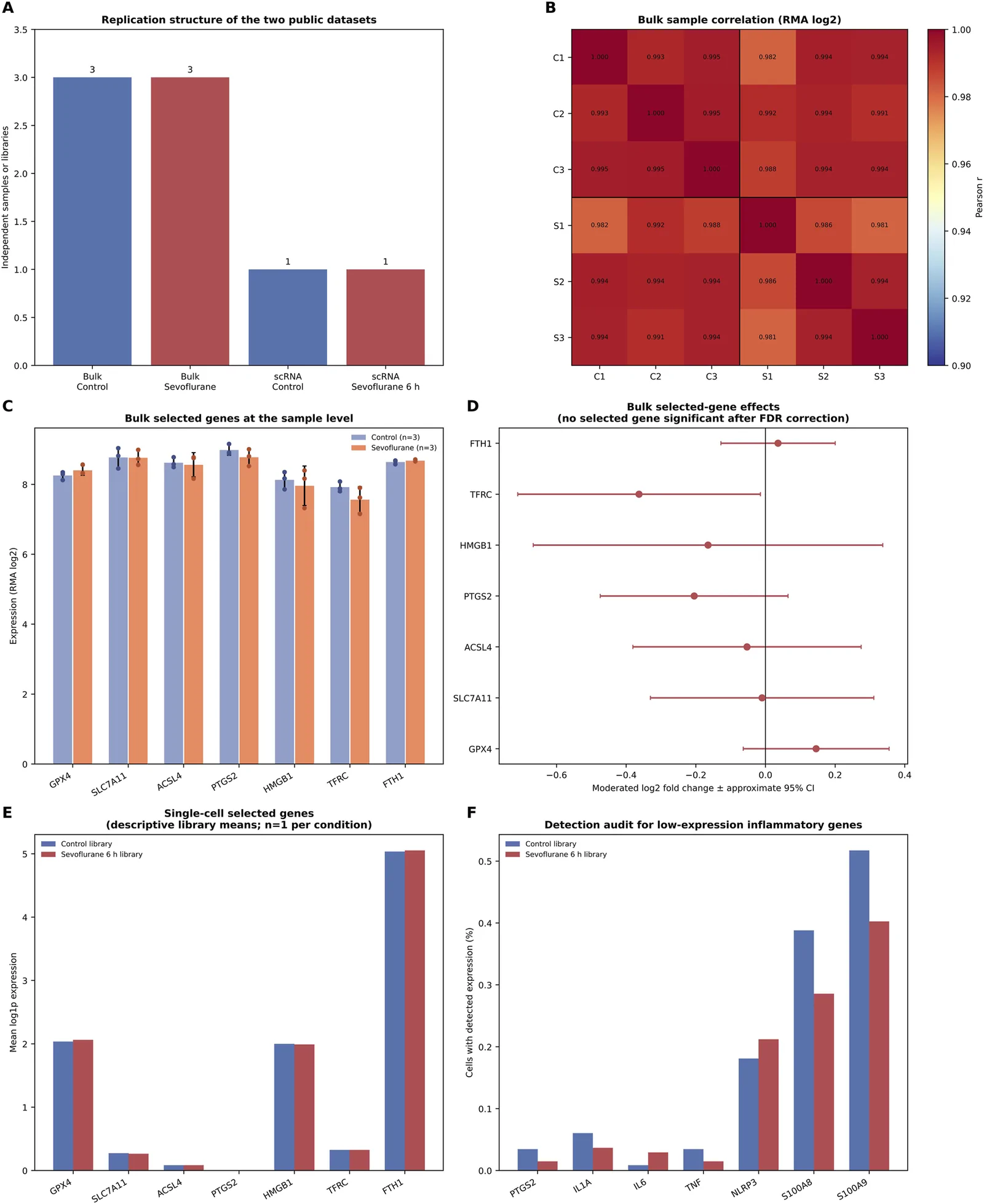
Replication and selected-gene audit across the two public datasets. **(A)** Number of independent bulk samples and single-cell libraries in the control and sevoflurane comparisons. **(B)** Pearson correlation heatmap of the six bulk hippocampus samples. **(C)** Sample-level expression of GPX4, SLC7A11, ACSL4, PTGS2, HMGB1, TFRC, and FTH1 in bulk hippocampus (bars, mean ± SD; points, individual samples). **(D)** Moderated log2 fold changes with approximate 95% confidence intervals for the selected bulk genes; none was significant after FDR correction. **(E)** Mean log1p expression of the selected genes in the single control and sevoflurane 6 h libraries; descriptive only. **(F)** Detection fraction for selected low-expression inflammatory genes in the two single-cell libraries.

### 3.9 *In vitro* validation of ferroptosis-related gene dysregulation after sevoflurane and H_2_O_2_ exposure

Quantitative RT-PCR in C6 glioma cells and HA astrocytes showed significant dysregulation of all four selected ferroptosis-related genes across Control, Sevo, H_2_O_2_, and Sevo + H_2_O_2_ groups (Figure 9). All three treatment groups differed from Control for each gene in both cell lines (Dunnett-adjusted P < 0.05). In C6 cells, relative GPX4 expression was 0.80, 0.67, and 0.43 in the Sevo, H_2_O_2_, and Sevo + H_2_O_2_ groups, SLC7A11 was 0.79, 0.62, and 0.36, ACSL4 was 1.27, 1.45, and 1.85, and PTGS2 was 1.31, 1.58, and 2.16. In HA astrocytes, relative GPX4 expression was 0.76, 0.60, and 0.35, SLC7A11 was 0.73, 0.56, and 0.31, ACSL4 was 1.30, 1.55, and 2.10, and PTGS2 was 1.35, 1.70, and 2.35, respectively (all Dunnett-adjusted P < 0.05 versus Control). These findings show consistent regulation of ferroptosis-related genes after both single and combined exposures in both cell systems. The opposing regulation of the protective markers GPX4/SLC7A11 and pro-ferroptotic markers ACSL4/PTGS2 is consistent with a ferroptosis-related transcriptional pattern. The matched single-agent groups indicate that both exposures contributed to the observed pattern, and the larger response in the combined group should not be interpreted as synergy or proof of ferroptotic cell death without consistent interaction effects and functional assays.

**FIGURE 9:**
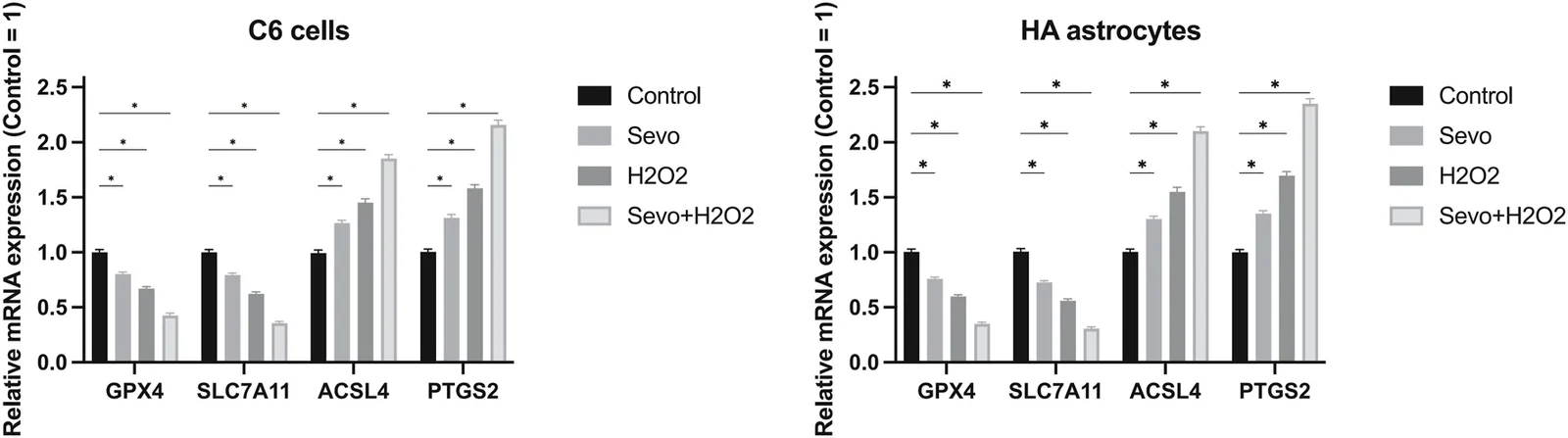
qRT-PCR analysis of ferroptosis-related gene expression after sevoflurane and H_2_O_2_ exposure. Relative expression of GPX4, SLC7A11, ACSL4, and PTGS2 in Control, Sevo (3.3% for 6 h), H_2_O_2_ (200 μM for 24 h), and Sevo + H_2_O_2_ groups in C6 glioma cells and HA astrocytes. *P < 0.05 versus Control; significant comparisons are displayed with a single asterisk. Data represent mean ± SEM from three independent experiments. Two-way ANOVA (sevoflurane × H_2_O_2_) was followed by Dunnett’s multiple-comparisons test against Control.

## 4 Discussion

This re-analysis yields a substantially more cautious interpretation than a model in which sevoflurane uniformly activates ferroptosis and downstream neuroinflammation. In the aged-rat hippocampal bulk dataset, no probe set reached FDR < 0.05 (minimum FDR = 0.071). In the human prefrontal cortex single-cell dataset, the single-library comparison was descriptive; canonical ferroptosis genes showed only small differences, predefined gene-set summaries were similar, and observed composition shifts remained below two percentage points. No formal cross-species correlation or joint significance test was used. Taken together, the public transcriptomic evidence is limited and non-confirmatory rather than supportive of a robust cross-system ferroptosis or neuroinflammation signature.

The negative or weak ferroptosis signal in our re-analysis does not imply that ferroptosis cannot occur after sevoflurane exposure. The original GSE141242 study reported broad transcriptional changes in aged rat hippocampus using its original analytical strategy (Wang et al., 2020), while the original GSE196239 study demonstrated transient and cell-type-dependent transcriptional responses in human embryonic prefrontal cortex cultures (Chang et al., 2023). In addition, direct experimental studies have reported sevoflurane-associated ferroptosis through H2O2/ATF3, PRKCD-Hippo, and PLIN4-Hippo pathways (Kang et al., 2024; Lv et al., 2024; Zeng et al., 2024), and a recent public-data re-analysis proposed an SLC7A11-centered mechanism (Hu et al., 2024). The discrepancy likely reflects differences in exposure intensity and duration, developmental stage, neural compartment, experimental platform, statistical thresholds, and the specific molecular criteria used to define ferroptosis. Our results therefore narrow the claim: these particular public datasets do not independently reproduce a strong coordinated ferroptosis transcriptional program under the analytical framework applied here.

The two transcriptomic systems should not be treated as formal cross-species validation. The bulk dataset represents hippocampus from 18-month-old rats *in vivo*, whereas the single-cell dataset represents human embryonic prefrontal cortex mixed-cell cultures. These systems differ simultaneously in species, brain region, developmental state, tissue architecture, and exposure context. Comparative single-cell studies have shown that broadly homologous human and mouse cortical cell classes can nevertheless display substantial species-specific differences in gene expression and cell-type proportions (Hodge et al., 2019). Accordingly, we evaluated whether conclusions were qualitatively compatible but did not assign inferential meaning to a cross-system correlation.

The single-cell statistical design imposes an additional limitation. GSE196239 contains one library for each experimental condition, so individual cells cannot be treated as independent biological replicates for condition-level inference. Methodological benchmarks have shown that cell-level differential expression tests that ignore biological replication can produce anti-conservative P values and false discoveries (Squair et al., 2021; Crowell et al., 2020). Pseudobulk or mixed-model approaches are preferred when multiple biological samples are available (Crowell et al., 2020), but a valid condition-level pseudobulk comparison is not possible with only one library per condition. For this reason, we did not use cell-level adjusted P values, cell-type DEG counts, or enrichment results to support condition-level claims. Interpretation was instead restricted to descriptive key-gene expression, predefined gene-set summaries, and observed library composition.

The *in vitro* experiment used a four-group exposure design. The design included Control, sevoflurane-only, H_2_O_2_-only, and combined Sevo + H_2_O_2_ groups. Both exposures changed ferroptosis-related gene expression in the same directions, and the combined group showed the largest magnitude. Nevertheless, gene-expression changes alone do not establish ferroptotic cell death or a pharmacologic interaction. In our experiment, GPX4 and SLC7A11 decreased, whereas ACSL4 and PTGS2 increased. Because GPX4 is a key ferroptosis suppressor and ACSL4 is a major determinant of ferroptosis sensitivity (Yang et al., 2014; Doll et al., 2017), this directional pattern is biologically consistent with ferroptosis-related regulation but is not definitive evidence of ferroptotic cell death. Instead, the qRT-PCR results provide complementary evidence of ferroptosis-related pathway perturbation after sevoflurane and H_2_O_2_ exposure.

Cell identity remained an important descriptive source of variation. Astrocytes support neural homeostasis and glutathione-dependent antioxidant defense (Dringen and Hirrlinger, 2003; Sofroniew and Vinters, 2010). Ferroptosis and inflammation gene-set values differed more across broad cell types than between the observed control and sevoflurane libraries. These patterns may help prioritize cell types for replicated experiments. However, the present data do not identify a uniquely ferroptosis-vulnerable population because the condition comparison lacks biological replicate libraries.

Several limitations should be emphasized. First, both transcriptomic datasets are small at the biological-sample level; the bulk cohort contains three animals per group and the single-cell dataset contains only one library per condition. Second, the datasets differ in species, age, brain region, and *in vivo* versus *in vitro* context, precluding formal joint inference. Third, transcript abundance alone cannot establish ferroptotic cell death, which ideally requires orthogonal evidence such as lipid peroxidation, iron dependence, characteristic ultrastructural changes, and rescue by ferroptosis inhibitors. Fourth, the qRT-PCR experiment included only three independent experiments per group and measured transcript abundance without lipid peroxidation, labile iron, viability, or rescue endpoints. Finally, the current transcriptomic analysis is a re-analysis of public data and should be considered hypothesis-generating.

These limitations are also informative. They show that strong mechanistic claims about a sevoflurane-ferroptosis-neuroinflammation cascade are not supported by the present public transcriptomic evidence alone. Future studies should use replicated direct sevoflurane exposure models, analyze sample-level rather than cell-level condition effects, incorporate ferroptosis-specific functional assays, and validate findings across independent human-relevant systems. Such designs will be necessary to distinguish a reproducible ferroptosis mechanism from broader oxidative and stress-response transcriptional changes.

## 5 Conclusion

This integrated re-analysis indicates that sevoflurane exposure is associated with transcriptional changes in the examined systems, but the available public datasets do not show a robust and reproducible ferroptosis or neuroinflammation signature. The aged-rat hippocampal dataset showed no FDR-significant probe sets, while the human single-cell dataset showed only small descriptive changes in canonical genes, predefined gene-set summaries, and observed composition with one library per condition. In contrast, sevoflurane and H_2_O_2_ each reduced GPX4/SLC7A11 and increased ACSL4/PTGS2 in C6 glioma cells and HA astrocytes, with the largest changes in the combined group, providing complementary evidence of ferroptosis-related pathway perturbation under both exposures. Although the directional gene pattern was compatible with ferroptosis-related regulation, these findings should not be interpreted as proof of ferroptotic cell death or synergy. Overall, the results support a cautious, hypothesis-generating interpretation and underscore the need for replicated direct-exposure studies with larger biological sample sizes, sample-level statistics, and orthogonal ferroptosis assays.

## Data Availability

The datasets presented in this study can be found in online repositories. The names of the repository/repositories and accession number(s) can be found in the article/supplementary material.
